# A Multilevel Model to Estimate the Within- and the Between-Center Components of the Exposure/Disease Association in the EPIC Study

**DOI:** 10.1371/journal.pone.0117815

**Published:** 2015-03-18

**Authors:** Francesco Sera, Pietro Ferrari

**Affiliations:** 1 Molecular and Nutritional Epidemiology Unit, Cancer Prevention and Research, ISPO, Florence, Italy; 2 Nutrition and Metabolism Section, International Agency for Research on Cancer, Lyon, France; IRCCS Istituto Oncologico Giovanni Paolo II, ITALY

## Abstract

In a multicenter study, the overall relationship between exposure and the risk of cancer can be broken down into a within-center component, which reflects the individual level association, and a between-center relationship, which captures the association at the aggregate level. A piecewise exponential proportional hazards model with random effects was used to evaluate the association between dietary fiber intake and colorectal cancer (CRC) risk in the EPIC study. During an average follow-up of 11.0 years, 4,517 CRC events occurred among study participants recruited in 28 centers from ten European countries. Models were adjusted by relevant confounding factors. Heterogeneity among centers was modelled with random effects. Linear regression calibration was used to account for errors in dietary questionnaire (DQ) measurements. Risk ratio estimates for a 10 g/day increment in dietary fiber were equal to 0.90 (95%CI: 0.85, 0.96) and 0.85 (0.64, 1.14), at the individual and aggregate levels, respectively, while calibrated estimates were 0.85 (0.76, 0.94), and 0.87 (0.65, 1.15), respectively. In multicenter studies, over a straightforward ecological analysis, random effects models allow information at the individual and ecologic levels to be captured, while controlling for confounding at both levels of evidence.

## Introduction

The relationship between aetiological factors and risk of disease in multicenter studies can be evaluated at the individual and at the aggregate (ecologic) levels [1–3], thus reflecting within- and between-center associations, respectively. In order to estimate simultaneously the two components of the exposure/disease association, multilevel models can be used [4]. In this framework, the role of (a possibly consistent list of) individual and aggregate level confounding variables can be accounted for. In addition, the use of random effects makes it possible to quantify the heterogeneity across centers of the association in multicenter studies, and to investigate the potential sources of such heterogeneity.

The characteristics of individual level and ecologic analyses have been extensively investigated [5–7]. Ecologic analysis is relatively efficient when the between-population exposure variability is large relative to the within-population variation [3, 8]. However, individual and ecologic analyses are prone to bias due to confounding [3]. Generally, individual level confounders are assessed through specifically designed questionnaires, e.g. dietary or lifestyle. Furthermore, over individual level studies, ecologic analysis may be more robust to classical (random) measurement errors but prone to systematic exposure misclassification [8].

Among the rationale that motivated the European Prospective Investigation into Cancer and Nutrition (EPIC), a large multicenter cohort study on diet and cancer conducted in ten Western European countries [9], was the idea of complementing aetiological evidence at the individual level with information available at the aggregate level through a comparison of dietary mean intakes with mean incidence rates [2]. In EPIC, cohorts from populations with diverse dietary exposures were combined, in order to increase between-subject variability and thereby reduce the impact of measurement errors of individual level dietary assessments [2]. In addition, substantial effort was invested in the collection of dietary measures harmonized across recruitment centers, by means of 24-hour dietary recall (24-HDR) [10]. 24-HDR measurements are used in linear regression calibration models [11] to correct for random and systematic errors in center-specific dietary questionnaire measurements [12, 13].

Multilevel analysis on survival data with random effects has been extensively described, including a gamma frailty model where the likelihood function was maximized by an EM algorithm [14], or a two-step algorithm to maximize a penalized partial likelihood [15], a proportional hazard models with random intercept and/or random slope that entails adaptation of penalized likelihood [16], Gibbs sampling [17, 18], or EM algorithm [19].

In this work a random effects piecewise exponential proportional hazards model was used to estimate the individual and aggregate components of the association between fiber intake and risk of colorectal cancer (CRC). This individual-level relationship was recently evaluated in the EPIC study using a standard approach [20]. A multilevel random effects model was used to estimate aggregate and individual-level components of the dietary fiber and CRC relationship, thus accounting for study center heterogeneity, both in terms of disease occurrence and exposure/disease associations [21, 22]. In order to correct for measurement errors, a linear regression calibration model was also employed [12]. The use of the random effects model is illustrated and discussed.

## Materials and Methods

The rationale and methodology used in the EPIC study have been previously described in detail [9]. Briefly, in the EPIC cohort study, participants were recruited from 28 centers in 10 European countries: France (North-East, North-West, South, South-coast), Italy (Ragusa, Naples, Florence, Turin, Varese), Spain (Granada, Murcia, Navarra, San Sebastian, Asturias), United Kingdom (Oxford Health conscious and General population, Norfolk), The Netherlands (Bilthoven and Utrecht), Greece, Germany (Heidelberg and Potsdam), Sweden (Malmo and Umeå), Denmark (Aarhus and Copenhagen) and Norway (South-East and North-West). Between 1992 and 1998, information on dietary, anthropometric and lifestyle factors was collected from a total of 521,457 subjects (70% women), mostly aged from 35 to 70 years. Written informed consent was provided by all study participants. Ethical approval for the EPIC study was provided from the review boards of the International Agency for Research on Cancer (IARC) and local participating centers. Information on habitual individual dietary intake was assessed using different validated dietary questionnaires across participating countries to capture geographical specificity of diet [9]. A single standardized 24-hour recall (24-HDR) of actual food consumption during the previous day for a large (7% of the whole study) representative sub-sample of the entire EPIC cohort was also collected [10]. Lifestyle questionnaires were used to assess additional information from study participants, including physical activity, lifetime alcohol use, smoking history, level of education and medical history.

In this work, after systematic exclusions of participants with missing, incomplete, or implausible information, as detailed elsewhere [20], a total of 478,461 subjects were included in the analysis. Out of 5,262,998 person-years accumulated over an average follow-up time of 11.0 years, 4,517 CRC cases were identified. Cancer incidence data were coded in accordance with the 10^th^ Revision of the International Classification of Diseases (ICD-10) and the second revision of the International Classification of Disease for Oncology (ICDO-2).

### Statistical analysis

#### Variability of exposure and confounders

The between- and within-center variability of dietary and non-dietary exposures were evaluated. For this purpose, variance components were estimated using a mixed model where the variable ‘center’ was modeled with a random effect, as detailed in S1 Appendix. Variance component terms were used to compute the intraclass correlation coefficient (ICC), which expresses the proportion of exposure heterogeneity at the center-level compared to total variability. The extent of between- and within-center variability was evaluated also for dichotomous exposure variables, and the associated ICC values.

#### The disease model

In this work, age was used as main the time scale variable, and it was assumed that the hazard function, *λ*(*t*), was piecewise constant in the *s* = 1,…,*S* age intervals, as

$$\lambda \left(t\right)=h_{0}\overset{S}{\underset{k=1}{\sum }}g_{k}I_{k}\left(t\right)\;,\;\text{with}\;I_{k}\left(t\right)=\;\{\begin{array}{c} \;1\;if\;\tau_{k-1}\leq t<\tau_{k}\\ 0\;\mathrm{elsewhere} \end{array}\;,$$

Where *h*_0_ is the baseline hazard, and *g*_1_ = 1,*g*_2_,*g*_3_,…,*g*_*S*_, are the relative hazard. We considered *S* = 7 age intervals, i.e. for the categories <49 years, 50–54, 55–59, 60–64, 65–69, 70–74, >75 years with first and last age intervals being open-ended to avoid a sparse number of CRC cases. The representation of a classical survival likelihood with a piecewise constant hazard by a Poisson likelihood is well known [23]. Thus, the time-to-event data becomes a counting process of 0 events or 1 event occurring with an offset of the time experienced at risk for the event within each time interval. Each study subject *i* = 1,…,*n*_*j*_ within center *j* = 1,…,*J* was represented by *S*_*i*_ ≤ *S* observations equal to the number of time intervals up to failure or censoring. For each subject, the intervals *S*_*i*_ ranged from the interval corresponding to age at entry (*S*_*i*,min_) to the interval corresponding to age at failure/censoring (*S*_*i*,max_). A variable indicating the presence of the event of interest was created, for interval *k* = *S*_*i*.min_,…,*S*_*i*,max_ as

$$d_{ijk}=0\;\text{for}\;k<S_{i,\text{max}}\;,\;\text{and}\;d_{ijk}=\{\begin{array}{c} \;1\;\mathrm{failure}\\ \;0\;\mathrm{censored} \end{array}\;,\;\text{for}\;k=S_{i,\text{max}}$$

A random effects model was fitted as

$$d_{ijk}∼\mathrm{Poisson}\left(\mu_{ijk}\right)$$

$$\text{ln}\left(\mu_{ijk}\right)=\text{ln}\left(t_{ijk}\right)+\alpha_{k}+\delta_{0j}+\delta_{1j}\cdot sex_{ij}+\left(X_{ij}-{\bar{X}}_{.j}\right)\beta_{Wj}+\left({\bar{X}}_{.j}-{\bar{X}}_{..}\right)\beta_{B}+\;+{\left(Z_{ij}-{\bar{Z}}_{.j}\right)}^{'}\gamma_{W}+{\left({\bar{Z}}_{.j}-{\bar{Z}}_{..}\right)}^{'}\gamma_{B}$$

$$\begin{array}{l} \delta_{0j}=\delta_{0}+u_{oj}\\ \delta_{1j}=\delta_{1}+u_{1j}\\ \beta_{Wj}=\beta_{1}+u_{Wj} \end{array}$$,

and

$$\left[\begin{array}{c} u_{0j}\\ u_{1j}\\ u_{Wj} \end{array}\right]∼MVN\left[\left(\begin{array}{c} 0\\ 0\\ 0 \end{array}\right),\left(\begin{array}{ccc} \sigma_{0}^{2} & \sigma_{01} & \sigma_{W,0}\\ \sigma_{01} & \sigma_{1}^{2} & \sigma_{W,1}\\ \sigma_{W,0} & \sigma_{W,1} & \sigma_{W}^{2} \end{array}\right)\right]$$

Indicated with *τ*_*k*_ and *τ*_*k−1*_, the extreme of age interval *k*, for *k* = *S*_*i*.min−1_,…,*S*_*i*,max−1_, the terms *t*_*ijk*_ are equal to 5 years, whereby for the first interval *k* = *S*_*i*,min_ and for the last interval *k* = *S*_*i*,max_, the terms *t*_*ijk*_ express the time unit spent in interval *k*, that is *t*_*ijk*_ = *τ*_*k*_ – age at entry_(*i*)_, and *t*_*ijk*_ = *τ*_*k*_ – age at exit_(*i*)_ – *τ*_*k*−1_, respectively. The terms *α*_*k*_ = ln(*g*_*k*_) indicate the logarithm of the relative hazard were estimated as coefficients of a list of indicator variables *h*_*ijk*_, i.e. one for each age interval except the category 60–64 years that was set as reference. The term δ_0*j*_ is a random effect that models the center effect on the logarithm of the baseline hazard among men. Sex was included as an indicator variable (0 = men, 1 = women), and modelled with a random effect coefficient δ_1*j*_. The variability of the outcome is captured by the term $\sigma_{0}^{2}$ in men, while in women it is equal to $\sigma_{0}^{2}+\sigma_{1}^{2}+2\sigma_{01}$. In this way, the disease model allows the variability of the outcome across centres to be sex-specific. A negative value of the covariance term *σ*_01_ permits the variability in women to be lower than in men.

The variable *X*_*ij*_ is the exposure factor of interest, ie. dietary fiber from questionnaire measurements in this work; the terms ${\bar{X}}_{.j}$ are the center-specific means, and **Z**_*ij*_ and ${\bar{Z}}_{.j}$ are vectors of confounding variables at the individual and aggregate levels, respectively. The coefficients (*β*_*W*_ and *β*_*B*_) model the relationship of dietary fiber and risk of colorectal cancer at the individual and aggregate levels, respectively. The terms *β*_*Wj*_ (random slope) have a fixed (*β*_*W*_) and a random (*u*_*wj*_) component, which capture heterogeneity of the exposure/disease associations across centers. The terms *σ*_*W*,0_ and *σ*_*W*,1_ express the covariance between random intercepts and random slope in men, as *σ*_*W*,0_ and women, as *σ*_*W*,0_ + *σ*_*W*,1_. In the case of a positive association, a positive (negative) covariance would suggest that the association between dietary fiber and CRC risk is more (less) pronounced in centers displaying higher CRC incidence. The evaluation of the heterogeneity of the exposure/risk of disease association across centers is of particular relevance in international collaborative studies. Unlike a fixed effects analysis, where this evaluation involves a large number of interaction terms, in a multilevel model, the variance associated to a random slope constitutes a succinct way to quantify the heterogeneity of associations. In addition, potential heterogeneity can be investigated in terms of aggregate level variables, thus exploring the extent of cross-level confounding in the association.The vector of coefficients, **γ**_*W*_ and **γ**_*B*_, model the relationship between confounding factors and cancer risk at the individual and aggregate levels, respectively. For the sake of simplicity, **γ**_*W*_ is assumed constant across centers, but this assumption could possibly be relaxed.

Adjustment for confounding variables was performed at the individual and at the aggregate level in the disease model. At the individual level the model included: red meat baseline alcohol consumption, energy from fat, energy from non-fat and non-alcohol sources, participant’s height and weight, physical activity (inactive/moderately inactive, moderately active/active, unknown), smoking habits (never, former/current, unknown) and educational level (no degree to secondary school, university degree, not specified/missing), consistently with a recent EPIC study [20].

At the aggregate level, models included center-specific means of energy from sources other than fat and alcohol, baseline alcohol consumption, latitude, the percentage of subjects with university degrees and the percentage of former and current smokers. Moreover, latitude of participating centers was considered to account for potential residual ecologic confounding.

In this work, models with different degrees of complexity have been fitted and compared. First, a model with sex-specific random intercept terms was estimated (model (1)). Then, individual level variables were included (model (2)). Aggregate level variables were added, after centering individual level variables to center-specific means (model (3)). Finally, a model with random slopes for the term $\left(X_{ij}-{\bar{X}}_{.j}\right)$ was fitted (model (4)). Each model was adjusted for individual- and aggregate- level confounding factors Z, as detailed in S2 Appendix.

For proper comparison of dietary measurements between cohorts, and to partially correct risk estimates for the effect of dietary measurement errors, a fixed-effect multivariate linear regression calibration model was used [11, 24], in which 24-hour dietary recall data from an 8% sample from all participating cohorts [25] were regressed on dietary questionnaire (DQ) measurements with adjustment for the same list of covariates mentioned above, and further controlled for the day of the week day and the season of recall measurements [12]. In a linear regression calibration model, the conditional expectations of true intake given the questionnaire measurements and the set of covariates, E(T|Q,Z), are computed, under the assumption that 24-hour dietary recall measurements are unbiased (11). E(T|Q,Z) values are used in the disease model. The variability of parameters was corrected to account for uncertainty due to the estimation process of regression calibration. Ten bootstrap samples were randomly generated, and the models (1) to (4) were fitted for each of them. The Rubin’s rule was used to obtain a correct estimate of the standard error by combining information across the ten different samples [26]. Linear regression calibration models were used for dietary fiber, red meat, energy from fat and energy from non-fat and non-alcohol sources. Alcohol intake was not corrected for measurement errors as the complexity of dealing with excess zero values in 24-HDRs due to the episodically consumption pattern of this factor, was beyond the scope of the present work.

The proportional hazards assumption was satisfied and evaluated via the inclusion into the disease model of interaction terms between dietary fiber and the time variable, t_ijk_ (data not shown). Statistical tests were two-sided. Multivariate linear regression calibration and piecewise exponential proportional hazards models were fitted using MLwiN [27] and Stata [28]. The syntax to carry out the different steps of the analysis is reported in S3 Appendix.

## Results

The center-specific frequencies of CRC cases, means and standard deviations of dietary fiber intake (g/day) using DQ measurements and after linear calibration are reported in Tables 1 and 2, for women and men respectively. In women, DQ dietary fiber means ranged from 18.7 g/day in Malmö to 27.0 g/day observed in Naples, and their ICC values were equal to 0.08 and 0.29 before and after linear calibration. In men, dietary fiber means went from 21.1 g/day in Malmo to values exceeding 30 g/day in the center of Murcia. ICC values were 0.12 and 0.30, before and after linear calibration. As previously observed and discussed in the EPIC study [29], linear regression calibration reduces the variability of the within-center component, thus resulting in larger ICC values.

**Table 1:** Centre-specific Frequency of Colorectal Cancer Cases (CRC), Person-Years (PY), and Age Standardized Incidence Ratio (SIR) and Associated 95%CI, and Centre-Specific Means of Dietary Fiber Intake (g/day) and Standard Deviation (SD), EPIC Original Data and After Linear Regression Calibration; Women.

						Dietary fiber			
						Original data		Linear calibration	
*Country* ^a^/Center	CRC	PY^b^	SIR	95%	CI	Mean	(SD)	Mean	(SD)
*Greece*	44	148.6	0.33	0.25,	0.44	20.4	(6.1)	17.3	(2.0)
*Spain*									
Granada	27	69.1	0.63	0.43,	0.92	20.0	(6.3)	17.8	(2.9)
Murcia	39	72.9	0.92	0.67,	1.26	25.9	(7.8)	24.0	(3.5)
Navarra	30	52.2	0.94	0.66,	1.35	22.3	(6.4)	19.0	(3.1)
San Sebastian	25	51.0	0.86	0.58,	1.28	23.2	(7.4)	21.8	(3.5)
Asturias	23	54.4	0.86	0.57,	1.29	20.6	(6.8)	19.2	(3.2)
*Italy*									
Ragusa	16	33.9	1.10	0.67,	1.79	24.0	(8.6)	18.2	(3.2)
Naples	28	56.4	0.78	0.54,	1.13	27.0	(7.2)	18.0	(2.9)
Florence	86	101.7	1.20	0.97,	1.48	21.1	(6.8)	20.3	(2.7)
Turin	41	50.2	1.19	0.87,	1.61	20.0	(6.5)	20.2	(2.6)
Varese	74	99.3	1.08	0.86,	1.36	19.4	(6.2)	18.7	(2.5)
*France*									
South coast of France	64	94.1	0.88	0.69,	1.12	23.1	(7.3)	20.6	(3.0)
South of France	116	177.4	0.89	0.74,	1.07	23.1	(7.1)	20.1	(3.0)
North-West of France	64	112.2	0.79	0.62,	1.01	22.7	(6.8)	18.9	(2.8)
North-East of France	179	315.6	0.81	0.70,	0.93	22.2	(6.8)	20.0	(2.8)
*Germany*									
Heidelberg	75	130.4	0.97	0.78,	1.22	19.6	(6.4)	19.7	(3.6)
Potsdam	97	141.7	1.19	0.97,	1.45	21.9	(6.3)	20.9	(3.4)
*The Netherlands*									
Bilthoven	52	137.2	0.90	0.69,	1.19	22.0	(5.8)	18.8	(5.2)
Utrecht	253	178.5	1.31	1.16,	1.48	21.9	(5.4)	21.8	(4.2)
*United Kingdom*									
Oxford Health conscious	181	385.4	0.94	0.82,	1.09	25.6	(9.3)	24.4	(4.2)
Oxford General population	32	58.2	0.75	0.53,	1.07	22.3	(7.6)	17.6	(3.5)
Cambridge	191	142.7	1.13	0.98,	1.31	22.4	(7.6)	16.8	(3.5)
*Denmark*									
Copenhagen	250	224.4	1.17	0.97	, 1.42	24.1	(8.0)	22.3	(5.1)
Aarhus	103	92.3	1.13	1.00	, 1.28	24.4	(7.8)	24.6	(5.0)
*Sweden*									
Malmo	219	187.4	1.02	0.89	, 1.17	18.7	(6.1)	15.4	(2.5)
Umeå	94	161.9	1.03	0.84	, 1.26	18.9	(6.8)	17.5	(2.6)
*Norway*									
South-East of Norway	117	188.8	1.42	1.19,	1.71	20.4	(6.1)	18.8	(2.8)
North-West of Norway	93	153.5	1.41	1.15,	1.73	20.7	(6.0)	18.8	(2.8)
All	2613	3,671.5				22.1	(7.3)	20.0	(4.2)
ICC^c^					0.08			0.29	

^a^ South-to-North

^b^ X 1,000

^c^ ICC = Intraclass correlation coefficient.

**Table 2:** Centre-specific Frequency of Colorectal Cancer Cases (CRC), Person-Years (PY), and Age Standardized Incidence Ratio (SIR) and Associated 95%CI, and Centre-Specific Means of Dietary Fiber Intake (g/day) and Standard Deviation (SD), EPIC Original Data and After Linear Regression Calibration; Men.

						Dietary fiber			
						Original data		Linear calibration	
*Country* ^a^/Center	CRC	PY^b^	SIR	95%	CI	Mean	(SD)	Mean	(SD)
*Greece*	61	99.1	0.48	0.37,	0.61	23.8	(7.0)	23.7	(3.0)
*Spain*									
Granada	29	30.9	0.79	0.50,	1.25	26.0	(8.2)	23.7	(4.7)
Murcia	18	21.1	0.97	0.69,	1.37	31.1	(9.0)	28.7	(5.2)
Navarra	33	33.3	0.85	0.63,	1.14	28.5	(7.9)	25.5	(4.6)
San Sebastian	43	49.0	1.24	0.97,	1.59	29.4	(9.0)	28.1	(5.3)
Asturias	62	48.7	1.05	0.73,	1.51	24.7	(7.5)	25.4	(4.7)
*Italy*									
Ragusa	21	31.6	0.85	0.56,	1.31	28.4	(9.2)	25.3	(4.1)
Naples	36	34.9	1.11	0.80,	1.55	23.8	(7.3)	27.4	(3.6)
Florence	89	69.7	1.33	1.08,	1.64	21.9	(7.0)	25.4	(3.4)
Turin	27	22.7	1.09	0.75,	1.60	22.0	(7.0)	24.0	(3.4)
Varese	61	99.1	0.48	0.37,	0.61	23.8	(7.0)	23.7	(3.0)
*France*									
South coast of France	-	-	-	-		-	-	-	-
South of France	-	-	-	-		-	-	-	-
North-West of France	-	-	-	-		-	-	-	-
North-East of France	-	-	-	-		-	-	-	-
*Germany*									
Heidelberg	127	112.9	1.02	0.86,	1.22	21.3	(7.2)	21.5	(4.4)
Potsdam	138	95.6	1.32	1.12,	1.56	24.1	(7.0)	22.9	(4.2)
*The Netherlands*									
Bilthoven	82	115.7	1.11	0.89,	1.38	26.1	(7.8)	26.2	(7.0)
Utrecht	-	-	-	-		-	-	-	-
*United Kingdom*									
Oxford Health c onscious	68	114.6	0.70	0.56,	0.89	27.1	(10.1)	30.8	(6.1)
Oxford general population	20	19.8	0.73	0.47,	1.13	22.2	(7.8)	21.5	(4.2)
Cambridge	236	117.7	1.11	0.98,	1.27	21.8	(7.6)	19.4	(4.7)
*Denmark*									
Copenhagen	346	196.1	1.16	1.04,	1.29	25.8	(8.5)	25.3	(5.4)
Aarhus	129	88.6	0.99	0.84,	1.18	26.1	(8.2)	27.2	(5.3)
*Sweden*									
Malmo	231	130.6	0.95	0.83,	1.08	21.1	(7.3)	17.6	(3.4)
Umeå	108	159.0	0.82	0.68,	0.99	21.2	(8.1)	20.4	(3.6)
*Norway*									
South-East of Norway	-	-	-	-		-	-	-	-
North-West of Norway	-	-	-	-		-	-	-	-
All	1,904	1,591.5				24.3	(8.4)	24.0	(5.9)
ICC^c^					0.12			0.30	

^a^ South-to-North

^b^ X 1,000

^c^ ICC = Intraclass correlation coefficient.

Means and frequencies of dietary and lifestyle variables at the aggregate level are reported in S1 Table and S2 Table. ICC values ranged from 8% (weight and the percentage of smokers) to 26% (height) in women, and from 3% (weight and the percentage of physically active subjects) to 25% (red meat intake) in men. The large ICC values observed for height (0.25 and 0.24, in women and men respectively) reflects the diversity of the EPIC populations.

The association between dietary fiber intake and CRC risk quantified by rate ratios (RR) estimated in a model using individual-level variables only (model (2) in S2 Appendix) was 0.90 (95% confidence interval (CI): 0.85, 0. 96) for a 10 g/d increment, as reported in Table 3. In a random effects model with dietary fiber and confounding factors included at the individual and aggregate levels (model (3)), RR were 0.90 (0.85, 0.96) and 0.85 (0.64, 1.14), for individual and aggregate levels respectively.

**Table 3:** Estimates of Rate Ratios (RR), 95%CI and Variance Components (VC) Obtained in Models (2) and (3), as Detailed in S2 Appendix, Using Individual and Aggregate Level Variables in the EPIC Study; Original data.

			Model (2)					Model (3)			
					VC					VC	
		RR	95% CI	Est^a^	(SE)	*P*-value	RR	95% CI	Est^a^	(SE)	*P*-value
*Intercept*											
Men				0.036	(0.016)	0.027			0.024	(0.012)	0.050
Women		0.66	0.58, 0.76	0.052	(0.018)	0.004	0.67	0.60, 0.76	0.028	(0.011)	0.014
*Individual level* ^b^											
Fiber	10 g/day	0.90	0.85, 0.96				0.90	0.85, 0.96			
Alcohol	15 g/day	1.06	1.04, 1.09				1.06	1.04, 1.09			
Red meat	100 g/day	1.04	0.94, 1.15				1.01	0.91, 1.11			
Energy from fat	125 Kcal/d	0.98	0.96, 1.00				0.99	0.97, 1.01			
Energy other sources^c^	125 Kcal/d	1.02	1.00, 1.04				1.02	1.00, 1.04			
Physical Activity											
(Moderately) Inactive		1.00	ref				1.00	ref			
(Moderately) Active		0.98	0.91, 1.04				0.99	0.93, 1.05			
Smoking status											
Non-smokers		1.00	ref				1.00	ref			
Smokers^d^		1.21	1.14, 1.29				1.20	1.12, 1.28			
*Aggregate level*											
Fiber	10 g/day						0.85	0.64; 1.14			
Alcohol	15 g/day						1.24	1.03, 1.49			
Energy other sources^c^	125 Kcal/d						1.11	1.03, 1.19			
% Graduates	1% increase						0.98	0.93, 1.03			
% Smokers^d^	1% increase						1.06	0.99, 1.12			
Latitude^e^	5° decrease						0.92	0.88, 0.97			

^a^ Variance component estimate

^b^ Also adjusted for weight, height and educational status

^c^ Energy from non-fat and non-alcohol sources

^d^ current and former smokers

^e^ Modelled as cosine(Latitude)

ref = reference category.

In models with a between-center random slope (model (4) in S2 Appendix, S3 Table), the fixed component of the coefficient was very similar to the RR estimate in model (3). The variance associated to the random component was 0.003 (SE = 0.004, p = 0.5). In men, the correlation between the intercept and the slope residuals was estimated as 0.30, suggesting that the protective association between fiber consumption and CRC was stronger in centers with elevated CRC incidence rates. Random slope models can be easily extended to test whether aggregate level variables explain some of the between-centers heterogeneity in the fiber/CRC association (model (4)). This can be achieved by making the random slope coefficient dependent on aggregate level variables in a random slope model [4]. However, in our study, there was limited heterogeneity across centers in the exposure/disease associations.

Multilevel models (1) to (4) were fitted with predicted values data after multivariate linear calibration models (Table 4 and S4 Table). The individual level coefficient of dietary fiber intake showed a more pronounced protective association with CRC risk, 0.85 (95%CI: 0.77, 0.93), (model (2)). The calibrated point estimates of individual and aggregate level fiber intake were equal to 0.85 (95%CI: 0.76, 0.94) and 0.87 (95%CI: 0.65, 1.15) respectively (Table 4), according to model (3). After linear calibration, the variance associated to the intercept random components were 0.020 (SE = 0.011) and 0.020 (SE = 0.007), in men and women respectively. Comparing model (3) with (2), random intercept variance components displayed a decrease equal to 47% and 62%, in men and women respectively.

**Table 4:** Estimates of Rate Ratios (RR), 95%CI and Variance Components (VC) Obtained in Models (2) and (3), as Detailed in S2 Appendix, Using Individual and Aggregate Level Variables in the EPIC Study; Linear Regression Calibration.

			Model (2)					Model (3)			
					VC					VC	
		RR	95% CI	Est^a^	(SE)	*P*-value	RR	95% CI	Est^a^	(SE)	*P*-value
*Intercept*											
Men				0.038	(0.017)	0.025			0.020	(0.011)	0.061
Women		0.66	0.57, 0.77	0.053	(0.019)	0.004	0.69	0.62, 0.77	0.020	(0.007)	0.005
*Individual level* ^b^											
Fiber	10 g/day	0.85	0.77, 0.93				0.85	0.76, 0.94			
Alcohol	15 g/day	1.06	1.04, 1.09				1.06	1.03, 1.09			
Red meat	100 g/day	1.05	0.89, 1.23				1.01	0.86, 1.19			
Energy from fat	125 Kcal/d	0.96	0.93, 1.00				0.97	0.94, 1.01			
Energy other sources^c^	125 Kcal/d	1.02	0.99, 1.06				1.01	0.97, 1.05			
Physical Activity											
(Moderately) Inactive		1.00	ref				1.00	ref			
(Moderately) Active		0.99	0.92, 1.06				1.00	0.93, 1.07			
Smoking status											
Non-smokers		1.00	ref				1.00	ref			
Smokers^d^		1.21	1.13, 1.29				1.19	1.12, 1.27			
*Aggregate level*											
Fiber	10 g/day						0.87	0.65, 1.15			
Alcohol	15 g/day						1.26	1.03, 1.54			
Energy other sources^c^	125 Kcal/d						1.16	1.06, 1.26			
% Graduates	1% increase						0.99	0.94, 1.04			
% Smokers^d^	1% increase						1.06	1.00, 1.13			
Latitude^e^	5° decrease						0.94	0.90, 0.98			

^a^ Variance component estimate

^b^ Also adjusted for weight, height and educational status

^c^ Energy from non-fat and non-alcohol sources

^d^ current and former smokers

^e^ Modelled as cosine(Latitude)

ref = reference category.

## Discussion

In this work, within- and between-center components of the association between dietary exposure and risk of disease were simultaneously estimated. A multilevel piecewise exponential proportional hazards model was used to complement standard evidence on the relationship between dietary fiber intake and risk of CRC at the individual level [20] with results obtained at the aggregate level. Results showed that dietary fiber intake was consistently negatively associated with the risk of CRC based on both levels of evidence.

It is very natural in the context of a multicenter study like EPIC to exploit and use each level of evidence to evaluate the relationship between dietary exposure and risk of disease. The use of information from the between-center component with a random effect model is based on the assumption that individual and aggregate components of the exposure/disease association are of similar magnitude. In the context of an etiological relationship that involves dietary factors, it is reasonable to assume that individual- and aggregate- level inference should provide consistent evidence, if aggregate-level confounding is adequately controlled. However, the two components can result in rather different estimates, possibly due to uncontrolled (residual) confounding and/or exposure measurement errors [8].

An important source of bias in ecological studies is the lack of information on confounding factors at the aggregate level [6]. In addition, confounding by center, typically arising from an uneven distribution of confounders across centers, was identified as one of the factors responsible of ecological bias or fallacy, that determines a difference between individual and ecologic risk estimates [5–7, 30, 31]. In the context of a multilevel model in a multicenter study, information is available at the aggregate level, thus potentially addressing both aggregate-level and individual-level confounding.

Inclusion of individual-level covariates explained an estimated 22% and 32% of the cross-center variability in CRC incidence, in women and men respectively (results obtained by comparing the variance components in models (2) and (1) in S2 Appendix), suggesting that some of the observed heterogeneity across center in CRC incidence was explained by individual level variables.

The association between alcohol consumption and CRC risk at the aggregate level was consistent with individuallevel associations observed in EPIC [32]. The percentage of subjects with university degrees and the percentage of former and current smokers were, respectively, negatively and positively associated with CRC risk. These variables are relevant determinants of unhealthy patterns of dietary and lifestyle factors [33, 34]. Overall, the association of confounders at the aggregate level was stronger than individual level counterparts. A variable expressing study center latitude was included in the random effects models, in an effort to explore potential residual confounding not captured by the variable center, for example exposure to vitamin D.

Our strategy to include potential individual-level confounding factors followed the standard practice of including in the model variables both associated to dietary fiber and the risk of colorectal cancer. The choice of aggregate level confounding followed a very similar strategy. We consistently used the same confounders at the individual and at the aggregate levels. In our study, a general principle of parsimony was used to include confounder factors at the aggregate level, mainly to account for the fact that aggregate level analysis does not carry the same amount of information, in terms of sample size, ie. statistical units. Means of energy from non-fat and non-alcohol sources, and center-specific percentages of former and current smokers displayed weak associations with dietary fiber (results not shown), but were related to CRC risk at the aggregate level.

In our working example, individual-level variables were centered on sex- and study center-specific means to make disease parameters readily interpretable as within- and between-center estimates of associations. As a result, individual- and aggregate- level variables were orthogonal, de facto limiting the possibility of evaluating cross-level confounding in our study. In addition, the fact that individual-level effect estimates for fiber intake were very similar in models (2) (exposure and confounders included as ‘overall’ variables) and (3) (exposure and confounders included as ‘centered’ variables) may suggest that the extent of cross-level confounding was limited in our study.

Cross-level interaction can be explored by relating the variability of association across centers in terms of aggregate-level variables. In this way, random slope coefficients that capture the association (between dietary fiber and risk of colorectal cancer in our study) at the individual level are dependent on aggregate-level variables [4]. The lack of heterogeneity across centers of exposure/disease associations in our study suggested there was little such variability to be explained.

In agreement with Sheppard and Prentice [8], the parameter of interest for biological inference, particularly in nutritional epidemiology, is the individual-level association of fiber with colorectal cancer risk (β_W_). The overall relationship between exposure and risk of disease was also fitted, in a model with no inclusion of the variable ‘center’. Neuhaus and Kalbfleisch pointed out that this model can be biased, if between- and the within-center associations are heterogeneous, with the degree of bias also depending on the degree of clustering of the exposure [35]. In this study, the between- and within-center estimates were of similar magnitude. The overall RR estimate was equal to 0.89 (95%CI: 0.85, 0.94), mostly reflecting the within-center component of the association. After linear calibration, dietary fiber showed a higher degree of clustering (ICC∼0.3), and the overall estimate was 0.87 (0.81, 0.94). It was noteworthy that the overall calibrated estimate displayed a gain in efficiency of 26% when compared to calibrated within-center estimates in model (3). This model did not include the possible increase in the standard errors due to clustering of the outcome, which was nevertheless low in our study.

Non-differential exposure misclassification may cause bias in either direction in multilevel models, depending upon the nature of the exposure (binary, or continuous) and the level of measurement, aggregate or individual [36]. DQ measurements are assumed to be affected by systematic misclassification, while the one single replicate of 24-HDR per subject are hypothesised to be relatively accurate but rather imprecise, as a result of random measurement errors [37, 38], possibly reflecting systematic misclassification and *day-to-day* within-person variability randomly distributed between subjects [39]. As a result, in multilevel studies in nutritional epidemiology, the aggregate level relationship between a dietary variable and an outcome may be biased by errors mostly originating from the lack of comparability of different dietary questionnaires across EPIC centers [2]. In addition, the accuracy of DQ measurements may have a geographical component in terms of accuracy. To minimize the effect of measurement errors in DQs (individual level) and to correct the lack of comparability of DQ measurements across countries (aggregate level), a multivariate linear regression calibration model was used [12].

In the multilevel model, both the within- and between-center components of the association between dietary fiber intake and CRC risk showed an inverse association, with no statistically significant difference between the two estimates. The slightly stronger relation observed at the aggregate level may suggest that information derived from between-population sources can be more robust to classical measurement errors than the corresponding individual level analysis [8]. After linear calibration, the within- and between-center components of the association were closer in magnitude. RR estimates for individual- and aggregate- level dietary fiber intake were 0.85 (0.76, 0.94) and 0.87 (0.65, 1.15), respectively.

In this study, the multivariable piecewise exponential proportional hazards random effects model showed that individual- and aggregate-level evidence were consistent in magnitude and direction, particularly after measurement error correction. The EPIC study offers an ideal design to explore the use of advanced statistical techniques, information on disease outcomes and dietary exposure(s) being available at different levels of evidence. In a relatively simple multilevel model these different components can ultimately become very informative.

## Supporting Information

S1 Appendix Calculation of Intraclass correlation coefficients. (DOCX)

S2 Appendix Multilevel models. (DOCX)

S3 Appendix Stata syntax for analysis. (DOCX)

S1 Table Center-Specific Means of Energy from Fat (En-fat) and Non-Fat and Non-alcohol Sources (E-NFNA), Red Meat Intake, Baseline Alcohol, Height, Weight, Percentage of Study Subjects (moderately) Active (PA), with University Degree (EDU), of (former and current) Smokers. Women.
(DOCX)

S2 Table Center-Specific Means of Energy from Fat (En-fat) and Non-Fat and Non-alcohol Sources (E-NFNA), Red Meat Intake, Baseline Alcohol, Height, Weight, Percentage of Study Subjects (moderately) Active (PA), with University Degree (EDU), of (former and current) Smokers. Men.
(DOCX)

S3 Table Estimates of Rate Ratios (RR), 95%CI and Variance Components (VC) Obtained in Models (1), and (4), as Detailed in Appendix B, Using Individual and Aggregate Level Variables in the EPIC Study. Original data.
(DOCX)

S4 Table Estimates of Rate Ratios (RR), 95%CI and Variance Components (VC) Obtained in Models (1), and (4), as Detailed in Appendix B, Using Individual and Aggregate Level Variables in the EPIC Study. Linear Regression Calibration.
(DOCX)
