# Supplementary material for: A Multilevel Model to Estimate the Within- and the Between-Center Components of the Exposure/Disease Association in the EPIC Study
Source: PLoS One. 2015 Mar 18;10(3):e0117815. doi: 10.1371/journal.pone.0117815 (PMC4365026; doi:10.1371/journal.pone.0117815)
Supplement: S1 Appendix — (DOCX) [file pone.0117815.s001.docx]

**Appendix S1. Calculation of Intraclass correlation coefficients.**

The continuous individual exposure level $C_{ij}$ for subjects $i=1,\ldots,n_{j}$ within center $j=1,\ldots,J$ was modeled as

$$C_{ij}=\alpha_{_{1j}}+\gamma_{1}\left(Z_{ij}\right)+\varepsilon_{1ij}$$

$$\alpha_{1j}=\alpha_{1}+u_{01j}$$

$u_{01j}$~$N\left(0,\sigma_{1}^{2}\right)$ and $\varepsilon_{1ij}$~ $N\left(0,\sigma_{2}^{2}\right)$.

The coefficient $\gamma_{1}$ models the effect of the variable(s) (Z) included as confounding factors, notably age at recruitment, while α_1_ is the average intercept term. The model has two random effects, $u_{01j}$ and $\varepsilon_{1ij}$, determining the variance components $\sigma_{1}^{2}$ and $\sigma_{2}^{2}$, which estimate between- and within-center variability. These terms are used to determine the intraclass correlation coefficient (ICC), as $\text{ICC}=\frac{\hat{\sigma}_{1}^{2}}{\hat{\sigma}_{1}^{2}+\hat{\sigma}_{2}^{2}}$, which estimates the proportion of exposure heterogeneity that is due to differences across centers. Overall and sex-specific models were used. For the overall ICC, clusters defined by both sex and center were used, ie. J=48 centers, 28 for women and 20 for men.

The extent of between- and within-center variability was also evaluated for dichotomous exposure variables, $D_{ij}$. A logistic random effect model was used, as

$D_{ij}\left|p_{ij}\right.$~$Bernoulli\left(p_{ij}\right)$

$$Logit\left(p_{ij}\right)=\alpha_{_{2j}}+\gamma_{2}\left(Z_{ij}\right)+\varepsilon_{2ij}$$

$$\alpha_{2j}=\alpha_{2}+u_{02j}$$

$u_{02j}$~$N\left(0,\sigma_{2,D}^{2}\right)$

In this context, the ICC was estimated using a latent variable approach, as $\text{ICC}=\frac{\hat{\sigma}_{2,D}^{2}}{\hat{\sigma}_{2,D}^{2}+\pi^{2}/3}$where $\pi^{2}/3$ is the variance of the logistic distribution of level-one residuals (ref: Snijders & Bosker, *Multilevel analysis: an introdction to basic and advanced multilevel modelling,* 1999). The observed binary response represents a continuous variable, whose observed values (0 and 1) are below or above a given threshold, which is a function of the set of covariates Z in the model. In a logit model, it is hypothesized that the underlying continuous variable follows a (standard) logistic distribution, whose variance is $\pi^{2}/3$. This quantity represents the level-1 variance, so that the ICC is estimated as the expression above.
