# Supplementary material for: A Multilevel Model to Estimate the Within- and the Between-Center Components of the Exposure/Disease Association in the EPIC Study
Source: PLoS One. 2015 Mar 18;10(3):e0117815. doi: 10.1371/journal.pone.0117815 (PMC4365026; doi:10.1371/journal.pone.0117815)
Supplement: S2 Appendix — (DOCX) [file pone.0117815.s002.docx]

**Appendix S2. Multilevel models.**

In this work, age was used as main the time scale variable, and it was assumed that the hazard function, $\lambda\left(t\right)$, was piecewise constant in the $s=1,\ldots,S$ age intervals, as

$\lambda\left(t\right)=h_{0}\sum_{k=1}^{S} g_{k}I_{k}(t)$, with $I_{k}\left(t\right)= \left\{\begin{matrix} 1 if \tau_{k-1}\leq t <\tau_{k} \\ 0 \text{ }elsewhere \end{matrix}\right.$ ,

where $h_{0}$ is the baseline hazard, and $g_{1}= 1, g_{2}, g_{3},\ldots, g_{S}$, are the relative hazards. We considered $S=7$ age intervals, i.e. for the categories <49 years, 50-54, 55-59, 60-64, 65-69, 70-74, >75 years with first and last age intervals being open-ended to avoid sparse number of CRC cases. Each study subject $i=1,\ldots,n_{j}$ within center $j=1,\ldots,J$ was represented by $S_{i}\leq S$ observations equal to the number of time intervals up to failure or censoring. For each subject, the intervals $S_{i}$ ranged from the interval corresponding to age at entry $\left(S_{i,\min}\right)$ to the interval corresponding to age at failure/censoring $\left(S_{i,\max}\right)$. A variable indicating the presence of the event of interest was created, for interval $k=S_{i.\min},\ldots,S_{i,\max}$ as

$d_{ijk}=0$ for $k<S_{i,\max}$, and $d_{ijk}=\left\{\begin{matrix} 1 failure \\ 0 censored \end{matrix}\right.$ , for $k=S_{i,\max}$

For $X$ and $Z$ expressing the main (dietary) exposure of interest and a vector of confounding variables, respectively, a sequence of random effects models were considered in this work:

- Model with random intercepts (1):

$d_{ijk}$~$Poisson\left(\mu_{ijk}\right)$

$$\ln\left(\mu_{ijk}\right)=\ln\left(t_{ijk}\right)+\alpha_{k}+\delta_{0j}+\delta_{1j}\cdot sex_{ij}$$

with $\delta_{0j}=\delta_{0}+u_{oj}$

$\delta_{1j}=\delta_{1}+u_{1j}$, and

$$\left[\begin{matrix} u_{0j} \\ u_{1j} \end{matrix}\right]\sim MVN\left[\left(\begin{matrix} 0 \\ 0 \end{matrix}\right),\left(\begin{matrix} \sigma_{0}^{2} & \sigma_{01} \\ \sigma_{01} & \sigma_{1}^{2} \end{matrix}\right)\right]$$

- Model with random intercepts and individual level variables (2):

$d_{ijk}$~$Poisson\left(\mu_{ijk}\right)$

$\ln\left(\mu_{ijk}\right)=\ln\left(t_{ijk}\right)+\alpha_{k}+\delta_{0j}+\delta_{1j}\cdot sex_{ij}+\left(X_{ij}-\overline{X}_{..}\right)\beta_{W}+\left(\mathbf{Z}_{ij}-\overline{\mathbf{Z}}_{..}\right)^{'}\mathbf{\gamma}_{W}$ with $\delta_{0j}=\delta_{0}+u_{oj}$

$\delta_{1j}=\delta_{1}+u_{1j}$, and

$$\left[\begin{matrix} u_{0j} \\ u_{1j} \end{matrix}\right]\sim MVN\left[\left(\begin{matrix} 0 \\ 0 \end{matrix}\right),\left(\begin{matrix} \sigma_{0}^{2} & \sigma_{01} \\ \sigma_{01} & \sigma_{1}^{2} \end{matrix}\right)\right]$$

Model with random intercepts and individual and aggregate level variables (3):

$d_{ijk}$~$Poisson\left(\mu_{ijk}\right)$

$$\ln\left(\mu_{ijk}\right)=\ln\left(t_{ijk}\right)+\alpha_{k}+\delta_{0j}+\delta_{1j}\cdot sex_{ij}+\left(X_{ij}-\overline{X}_{.j}\right)\beta_{W}+\left(\overline{X}_{.j}-\overline{X}_{..}\right)\beta_{B}+$$

$$+\left(\mathbf{Z}_{ij}-\overline{\mathbf{Z}}_{.j}\right)^{'}\mathbf{\gamma}_{W}+\left(\overline{\mathbf{Z}}_{.j}-\overline{\mathbf{Z}}_{..}\right)^{'}\mathbf{\gamma}_{B}$$

$$\delta_{0j}=\delta_{0}+u_{oj}$$

$$\delta_{1j}=\delta_{1}+u_{1j}$$

and $\left[\begin{matrix} u_{0j} \\ u_{1j} \end{matrix}\right]\sim MVN\left[\left(\begin{matrix} 0 \\ 0 \end{matrix}\right),\left(\begin{matrix} \sigma_{0}^{2} & \sigma_{01} \\ \sigma_{01} & \sigma_{1}^{2} \end{matrix}\right)\right]$

- Model with random intercept, individual and aggregate level variables, with random slopes (4):

$d_{ijk}$~$Poisson\left(\mu_{ijk}\right)$

$$\ln\left(\mu_{ijk}\right)=\ln\left(t_{ijk}\right)+\alpha_{k}+\delta_{0j}+\delta_{1j}\cdot sex_{ij}+\left(X_{ij}-\overline{X}_{.j}\right)\beta_{Wj}+\left(\overline{X}_{.j}-\overline{X}_{..}\right)\beta_{B}+$$

$$+\left(\mathbf{Z}_{ij}-\overline{\mathbf{Z}}_{.j}\right)^{'}\mathbf{\gamma}_{W}+\left(\overline{\mathbf{Z}}_{.j}-\overline{\mathbf{Z}}_{..}\right)^{'}\mathbf{\gamma}_{B}$$

$$\delta_{0j}=\delta_{0}+u_{oj}$$

$$\delta_{1j}=\delta_{1}+u_{1j}$$

$\beta_{Wj}=\beta_{1}+u_{Wj}$, and

$$\left[\begin{matrix} u_{0j} \\ u_{1j} \\ u_{Wj} \end{matrix}\right]\sim MVN\left[\left(\begin{matrix} 0 \\ 0 \\ 0 \end{matrix}\right),\left(\begin{matrix} \sigma_{0}^{2} & \sigma_{01} & \sigma_{W,0} \\ \sigma_{01} & \sigma_{1}^{2} & \sigma_{W,1} \\ \sigma_{W,0} & \sigma_{W,1} & \sigma_{W}^{2} \end{matrix}\right)\right]$$

This random slopes model can be easily extended to test whether effect modification by aggregate-level variables might explain some of the heterogeneity among centers’ slopes, for example by making center-specific regression coefficients dependent variables, as $\beta_{Wj}=\beta_{1}+\beta_{2}\left(\overline{\mathbf{Z}}_{.j}-\overline{\mathbf{Z}}_{..}\right)+u_{_{1j}}$. This parameterization can be shown to be equivalent to a model where $\beta_{2}$ is an interaction term between $\left(X_{ij}-\overline{X}_{.j}\right)$ and $\left(\overline{\mathbf{Z}}_{.j}-\overline{\mathbf{Z}}_{..}\right)$ in a random slope model.
