# Supplementary material for: A Multilevel Model to Estimate the Within- and the Between-Center Components of the Exposure/Disease Association in the EPIC Study
Source: PLoS One. 2015 Mar 18;10(3):e0117815. doi: 10.1371/journal.pone.0117815 (PMC4365026; doi:10.1371/journal.pone.0117815)
Supplement: S3 Appendix — (DOCX) [file pone.0117815.s003.docx]

**Appendix S3. Stata syntax for analysis.**

This is Stata syntax to carry out the different steps of the statistical analysis.

****** stset of data

stset *agexit*, failure(*outcome*) enter(*agenter*) origin(time 0) id(*id*)

****** split the dataset by agebands

stsplit *year*, at(15 (5) 105)

replace *outcome* =0 if *outcome* ==.

recode *year* 15 20 25 30 35 40=45

recode *year* 100 95 90 85 80=75

gen *tijk*=*_t* -_*t0*

****** aggregate data before analysis

collapse (sum) *cons* *y* *outcome*, by(*center* *exposure* *exposure_within* *exposure_between* *sex* *confounder* *confounder_within* *confounder_between* *id* *year* *_Iyear_45 _Iyear_50 _Iyear_55 _Iyear_65 _Iyear_70 _Iyear_75*)

describe

gen *lntijk*=ln(*tijk*)

gen *beta0*=1

gen *idobs*=_n

gen *idrec*=sum(1)

* Here Stata uses the command ‘runmlwin’. This is a Stata command which allows Stata users to run the powerful MLwiN multilevel modelling software from within Stata.runs MlWin

****** model (1)

runmlwin *outcome beta0 _Iyear_50 _Iyear_55 _Iyear_60 _Iyear_65 _Iyear_70 _Iyear_75 sex*, level2( *center* : *beta0 sex*) level1(*idobs*) discrete(distribution(poisson) offset( *lntijk* ) pql2) rigls mlwinpath(C:\Program Files (x86)\MLwiN v2.26\i386\MLwiN.exe) mlwinsettings(optimat) nopause

****** model (2)

runmlwin *outcome beta0 _Iyear_45 _Iyear_50 _Iyear_55 _Iyear_65 _Iyear_70 _Iyear_75 sex* *exposure* *confounder*, level2(*centre* : *beta0 sex*) level1(*idobs*) discrete(distribution(poisson) offset(*lntijk*) pql2) rigls mlwinpath(C:\Program Files (x86)\MLwiN v2.26\i386\MLwiN.exe) mlwinsettings(optimat) nopause

*model (3)*

runmlwin *outcome beta0 _Iyear_45 _Iyear_50 _Iyear_55 _Iyear_65 _Iyear_70 _Iyear_75 sex* *exposure_within exposure_between* *confounder_within confounder_between*, level2(*centre* : *beta0 sex*) level1(*idobs*) discrete(distribution(poisson) offset(*lntijk* ) pql2) rigls mlwinpath(C:\Program Files (x86)\MLwiN v2.26\i386\MLwiN.exe) mlwinsettings(optimat) nopause

*model (4)*

runmlwin *outcome beta0 _Iyear_45 _Iyear_50 _Iyear_55 _Iyear_65 _Iyear_70 _Iyear_75 sex* *exposure_within exposure_between* *confounder_within confounder_between*, level2(*centre* : *beta0 sex* *exposure_within*) level1(*idobs*) discrete(distribution(poisson) offset(*lntijk*) pql2) rigls mlwinpath(C:\Program Files (x86)\MLwiN v2.26\i386\MLwiN.exe) mlwinsettings(optimat) nopause

Further instructions are available upon request to the corresponding Author (ferrarip@iarc.fr).
