# Supplementary material for: A Multilevel Model to Estimate the Within- and the Between-Center Components of the Exposure/Disease Association in the EPIC Study
Source: PLoS One. 2015 Mar 18;10(3):e0117815. doi: 10.1371/journal.pone.0117815 (PMC4365026; doi:10.1371/journal.pone.0117815)
Supplement: S1 Table — Women. (DOCX) [file pone.0117815.s004.docx]

Table S1. Center-Specific Means of Energy from Fat (En-fat) and Sources Other Than Fat and Alcohol (E-NFNA), Red Meat Intake, Baseline Alcohol, Height, Weight, Percentage of Study Subjects (moderately) Active (PA), with University Degree (EDU), of former and current Smokers. **Women.**

| Country | Center | En-fat (kcal/day) | En-NFNA  (kcal/day) | Red meat (g/day) | Alcohol (g/day) | Height (cm) | Weight  (kg) | % PA | % EDU | % smokers |
| --- | --- | --- | --- | --- | --- | --- | --- | --- | --- | --- |
| Greece | - | 834.3 | 996.8 | 46.5 | 3.3 | 156.4 | 69.8 | 80.3 | 15.1 | 31.3 |
| Spain | Granada | 596.6 | 1043.8 | 16.3 | 1.6 | 155.6 | 70.7 | 86.7 | 9.3 | 22.5 |
|  | Murcia | 743.8 | 1268.0 | 15.1 | 4.9 | 156.3 | 69.2 | 78.6 | 12.3 | 27.1 |
|  | Navarra | 796.2 | 1156.0 | 50.2 | 4.0 | 157.3 | 68.1 | 82.7 | 7.7 | 32.2 |
|  | San Sebastian | 667.1 | 1177.7 | 46.4 | 6.8 | 158.4 | 67.6 | 76.7 | 7.7 | 30.9 |
|  | Asturias | 624.7 | 1129.1 | 34.8 | 4.1 | 156.8 | 68.8 | 81.9 | 9.9 | 32.7 |
| Italy | Ragusa | 723.5 | 1387.8 | 24.0 | 4.1 | 157.5 | 64.7 | 72.8 | 17.5 | 44.9 |
|  | Naples | 787.1 | 1535.4 | 50.8 | 8.0 | 156.7 | 66.4 | 54.5 | 16.6 | 58.3 |
|  | Florence | 747.5 | 1345.0 | 57.6 | 10.2 | 159.9 | 64.7 | 66.5 | 17.4 | 52.2 |
|  | Turin | 736.5 | 1185.2 | 45.8 | 9.3 | 159.0 | 62.6 | 68.1 | 10.9 | 43.1 |
|  | Varese | 750.4 | 1277.2 | 48.1 | 8.5 | 158.0 | 63.7 | 74.1 | 6.9 | 35.4 |
| France | South coast of France | 774.9 | 1268.6 | 48.2 | 11.3 | 161.2 | 59.5 | 26.1 | 38.1 | 39.0 |
|  | South of France | 774.5 | 1281.9 | 49.1 | 10.1 | 161.3 | 59.5 | 24.2 | 36.7 | 38.2 |
|  | North-West of France | 813.9 | 1310.8 | 51.5 | 10.9 | 161.0 | 59.8 | 23.0 | 31.8 | 37.7 |
|  | North-East of France | 796.3 | 1276.5 | 55.1 | 11.3 | 161.7 | 60.8 | 22.1 | 39.1 | 39.7 |
| Germany | Heidelberg | 627.5 | 1049.4 | 11.9 | 10.8 | 163.9 | 68.0 | 61.3 | 24.6 | 49.9 |
|  | Potsdam | 685.6 | 1165.1 | 10.2 | 8.2 | 162.9 | 68.4 | 50.1 | 28.8 | 39.5 |
| The Netherlands | Bilthoven | 687.2 | 1221.9 | 64.1 | 7.7 | 165.7 | 67.7 | 70.9 | 21.3 | 62.4 |
|  | Utrecht | 611.7 | 1121.1 | 58.4 | 9.0 | 164.3 | 68.7 | 75.1 | 16.1 | 57.4 |
| United Kingdom | Oxford Health conscious | 620.1 | 1204.5 | 9.1 | 8.0 | 164.2 | 63.2 | 49.8 | 51.1 | 37.2 |
|  | Oxford General population | 689.3 | 1258.4 | 39.8 | 7.3 | 162.4 | 67.2 | 52.4 | 38.8 | 45.3 |
|  | Cambridge | 656.3 | 1233.1 | 38.6 | 5.7 | 161.0 | 66.9 | 63.5 | 11.1 | 54.1 |
| Denmark | Copenhagen | 641.2 | 1193.2 | 71.8 | 14.6 | 164.2 | 69.0 | 41.0 | 11.4 | 57.2 |
|  | Aarhus | 660.8 | 1211.0 | 79.0 | 11.6 | 164.0 | 68.5 | 43.3 | 7.5 | 54.7 |
| Sweden | Malmo | 727.7 | 1182.4 | 28.1 | 7.4 | 163.6 | 67.0 | 46.1 | 23.6 | 54.3 |
|  | Umeå | 527.1 | 1060.1 | 2.0 | 1.8 | 164.4 | 66.4 |  | 24.3 | 39.8 |
| Norway | South-East of Norway | 544.8 | 1056.9 | 6.3 | 3.2 | 167.0 | 67.7 |  | 13.4 | 71.6 |
|  | North-West of Norway | 558.7 | 1100.5 | 6.3 | 2.3 | 166.2 | 67.7 |  | 11.4 | 72.0 |
|  | All | 684.1 | 1190.1 | 36.2 | 7.9 | 162.3 | 65.7 | 52.0 | 23.4 | 46.7 |
|  | ICC^a^ | 0.13 | 0.11 | 0.17 | 0.09 | 0.26 | 0.08 | 0.13 | 0.10 | 0.08 |

^a^ ICC=Intraclass correlation coefficient
