# Supplementary material for: A Multilevel Model to Estimate the Within- and the Between-Center Components of the Exposure/Disease Association in the EPIC Study
Source: PLoS One. 2015 Mar 18;10(3):e0117815. doi: 10.1371/journal.pone.0117815 (PMC4365026; doi:10.1371/journal.pone.0117815)
Supplement: S2 Table — Men. (DOCX) [file pone.0117815.s005.docx]

Table S2. Center-Specific Means of Energy from Fat (En-fat) and Sources Other Than Fat and Alcohol (E-NFNA), Red Meat Intake, Baseline Alcohol, Height, Weight, Percentage of Study Subjects (moderately) Active (PA), with University Degree (EDU), of former and current Smokers. **Men.**

| Country | Center | En-fat (kcal/day) | En-NFNA  (kcal/day) | Red meat (g/day) | Alcohol (g/day) | Height (cm) | Weight  (kg) | % PA | % EDU | % smokers |
| --- | --- | --- | --- | --- | --- | --- | --- | --- | --- | --- |
| Greece | - | 995.3 | 1196.8 | 68.6 | 18.1 | 170.0 | 80.6 | 38.6 | 19.9 | 80.8 |
| Spain | Granada | 839.7 | 1366.3 | 35.2 | 16.4 | 168.0 | 81.6 | 34.9 | 23.0 | 71.8 |
|  | Murcia | 899.5 | 1551.3 | 28.5 | 22.7 | 168.0 | 80.2 | 34.0 | 22.5 | 75.1 |
|  | Navarra | 1017.7 | 1480.0 | 76.9 | 33.1 | 169.0 | 82.1 | 38.5 | 11.4 | 68.4 |
|  | San Sebastian | 878.0 | 1542.6 | 73.1 | 32.5 | 170.2 | 80.9 | 47.6 | 9.8 | 69.1 |
|  | Asturias | 771.2 | 1396.0 | 59.6 | 28.1 | 168.8 | 81.2 | 40.7 | 15.4 | 71.5 |
| Italy | Ragusa | 859.1 | 1829.9 | 41.4 | 13.7 | 169.2 | 78.7 | 32.1 | 18.0 | 77.3 |
|  | Naples | 866.0 | 1715.8 | 71.7 | 26.0 | 173.3 | 78.9 | 31.7 | 14.8 | 75.5 |
|  | Florence | 828.2 | 1484.2 | 62.6 | 26.5 | 172.1 | 76.5 | 30.1 | 14.8 | 74.0 |
|  | Turin | 862.4 | 1603.6 | 64.4 | 29.2 | 171.3 | 77.7 | 36.4 | 7.6 | 72.4 |
|  | Varese | 995.3 | 1196.8 | 68.6 | 18.1 | 170.0 | 80.6 | 38.6 | 19.9 | 80.8 |
| France | South coast of France | - | - | - | - | - | - | - | - | - |
|  | South of France | - | - | - | - | - | - | - | - | - |
|  | North-West of France | - | - | - | - | - | - | - | - | - |
|  | North-East of France | - | - | - | - | - | - | - | - | - |
| Germany | Heidelberg | 755.6 | 1284.6 | 36.3 | 25.7 | 175.9 | 83.4 | 34.9 | 36.8 | 67.6 |
|  | Potsdam | 868.1 | 1427.2 | 31.8 | 22.5 | 174.9 | 82.7 | 44.3 | 49.4 | 67.7 |
| The Netherlands | Bilthoven | 894.8 | 1558.0 | 82.7 | 18.3 | 178.4 | 81.1 | 54.1 | 27.1 | 69.6 |
|  | Utrecht | - | - | - | - | - | - | - | - | - |
| United Kingdom | Oxford Health conscious | 714.1 | 1327.9 | 10.4 | 13.8 | 176.8 | 76.5 | 39.0 | 64.5 | 46.2 |
|  | Oxford General population | 755.6 | 1355.1 | 38.7 | 15.6 | 176.0 | 81.5 | 42.1 | 60.0 | 57.7 |
|  | Cambridge | 750.9 | 1337.4 | 41.5 | 11.4 | 174.0 | 79.4 | 49.9 | 13.7 | 77.3 |
| Denmark | Copenhagen | 843.0 | 1444.9 | 93.4 | 30.0 | 176.9 | 83.1 | 42.7 | 29.9 | 74.7 |
|  | Aarhus | 869.3 | 1442.4 | 95.6 | 23.9 | 176.4 | 82.8 | 41.2 | 25.0 | 74.5 |
| Sweden | Malmo | 968.8 | 1483.7 | 49.1 | 14.3 | 176.4 | 80.8 | 23.3 | 22.6 | 71.7 |
|  | Umeå | 753.6 | 1362.5 | 7.0 | 4.8 | 178.0 | 80.4 | - | 18.4 | 44.4 |
| Norway | South-East of Norway | - | - | - | - | - | - | - | - | - |
|  | North-West of Norway | - | - | - | - | - | - | - | - | - |
|  | All | 844.5 | 1419.8 | 54.1 | 20.3 | 174.7 | 80.8 | 39.6 | 27.2 | 68.4 |
|  | ICC^a^ | 0.09 | 0.12 | 0.25 | 0.11 | 0.24 | 0.03 | 0.03 | 0.10 | 0.08 |

^a^ ICC=Intraclass correlation coefficient.
