# Supplementary material for: A Multilevel Model to Estimate the Within- and the Between-Center Components of the Exposure/Disease Association in the EPIC Study
Source: PLoS One. 2015 Mar 18;10(3):e0117815. doi: 10.1371/journal.pone.0117815 (PMC4365026; doi:10.1371/journal.pone.0117815)
Supplement: S3 Table — Original data. (DOCX) [file pone.0117815.s006.docx]

Table S3. Estimates of Rate Ratios (RR), 95%CI and Variance Components (VC) Obtained in Models (1), and (4), as Detailed in Appendix B, Using Individual and Aggregate Level Variables in the EPIC Study.

|  |  |  | Model (1) |  |  |  |  |  |  | Model (4) |  |  |  |
| --- | --- | --- | --- | --- | --- | --- | --- | --- | --- | --- | --- | --- | --- |
|  |  |  |  |  | VC |  |  |  |  |  |  | VC |  |
|  |  | RR | 95% CI | Est^a^ | (SE) | *P*-value |  |  | RR | 95% CI | Est^a^ | (SE) | *P*-value |
| *Intercept* |  |  |  |  |  |  |  |  |  |  |  |  |  |
| Men |  |  |  | 0.053 | (0.022) | 0.016 |  |  |  |  | 0.023 | (0.012) | 0.051 |
| Woman |  | 0.64 | 0.56, 0.75 | 0.067 | (0.022) | 0.002 |  |  | 0.67 | 0.60, 0.76 | 0.026 | (0.008) | 0.002 |
|  |  |  |  |  |  |  |  |  |  |  |  |  |  |
| *Individual level*^b^ |  |  |  |  |  |  |  |  |  |  |  |  |  |
| Dietary fiber | 10 g/day |  |  |  |  |  |  |  | 0.90 | 0.85, 0.96 | 0.003^e^ | (0.004) | 0.486 |
| Alcohol | 15 g/day |  |  |  |  |  |  |  | 1.06 | 1.03, 1.09 |  |  |  |
| Red meat | 100 g/day |  |  |  |  |  |  |  | 1.01 | 0.91, 1.11 |  |  |  |
| Energy from fat | 125 Kcal/d |  |  |  |  |  |  |  | 0.99 | 0.97, 1.01 |  |  |  |
| Energy other sources^c^ | 125 Kcal/d |  |  |  |  |  |  |  | 1.02 | 1.00, 1.04 |  |  |  |
| Physical Activity |  |  |  |  |  |  |  |  |  |  |  |  |  |
| (Moderately) Inactive |  |  |  |  |  |  |  |  |  | ref |  |  |  |
| (Moderately) Active |  |  |  |  |  |  |  |  | 0.99 | 0.93, 1.05 |  |  |  |
| Smoking status |  |  |  |  |  |  |  |  |  |  |  |  |  |
| Non-smokers |  |  |  |  |  |  |  |  |  | ref |  |  |  |
| Smokers^d^ |  |  |  |  |  |  |  |  | 1.20 | 1.12, 1.28 |  |  |  |
|  |  |  |  |  |  |  |  |  |  |  |  |  |  |
| *Aggregate level* |  |  |  |  |  |  |  |  |  |  |  |  |  |
| Dietary fiber | 10 g/day |  |  |  |  |  |  |  | 0.86 | 0.65; 1.15 |  |  |  |
| Alcohol | 15 g/day |  |  |  |  |  |  |  | 1.26 | 1.05, 1.52 |  |  |  |
| Energy other sources^c^ | 125 Kcal/d |  |  |  |  |  |  |  | 1.10 | 1.03, 1.18 |  |  |  |
| % Graduate | 1% increase |  |  |  |  |  |  |  | 0.98 | 0.93, 1.03 |  |  |  |
| % Smokers^d^ | 1% increase |  |  |  |  |  |  |  | 1.06 | 0.99, 1.12 |  |  |  |
| Latitude^f^ | 5° decrease |  |  |  |  |  |  |  | 0.92 | 0.88, 0.97 |  |  |  |
|  |  |  |  |  |  |  |  |  |  |  |  |  |  |

^a^ Variance component estimate; ^b^ Also adjusted for weight, height and educational status; ^c^ Energy from sources other than fat and alcohol; ^d^ current and former smokers; ^e^ log-RR scale; ^f^ Modelled as cosine(Latitude); ref=reference category.
