# Supplementary material for: A Multilevel Model to Estimate the Within- and the Between-Center Components of the Exposure/Disease Association in the EPIC Study
Source: PLoS One. 2015 Mar 18;10(3):e0117815. doi: 10.1371/journal.pone.0117815 (PMC4365026; doi:10.1371/journal.pone.0117815)
Supplement: S4 Table — Linear Regression Calibration. (DOCX) [file pone.0117815.s007.docx]

Table S4. Estimates of Rate Ratios (RR), 95%CI and Variance Components (VC) Obtained in Models (1), and (4), as Detailed in Appendix B, Using Individual and Aggregate Level Variables in the EPIC Study. After Linear Regression Calibration.

|  |  |  | Model (1) |  |  |  |  |  |  | Model (4) |  |  |  |
| --- | --- | --- | --- | --- | --- | --- | --- | --- | --- | --- | --- | --- | --- |
|  |  |  |  |  | VC |  |  |  |  |  |  | VC |  |
|  |  | RR | 95% CI | Est^a^ | (SE) | *P*-value |  |  | RR | 95% CI | Est^a^ | (SE) | *P*-value |
| *Intercept* |  |  |  |  |  |  |  |  |  |  |  |  |  |
| Men |  |  |  | 0.053 | (0.022) | 0.016 |  |  |  |  | 0.020 | (0.011) | 0.060 |
| Woman |  | 0.64 | 0.56, 0.75 | 0.067 | (0.022) | 0.002 |  |  | 0.69 | 0.61, 0.77 | 0.019 | (0.007) | 0.005 |
|  |  |  |  |  |  |  |  |  |  |  |  |  |  |
| *Individual level*^b^ |  |  |  |  |  |  |  |  |  |  |  |  |  |
| Dietary fiber | 10 g/day |  |  |  |  |  |  |  | 0.85 | 0.76, 0.94 | 0.0008^e^ | (0.009) | 0.926 |
| Alcohol | 15 g/day |  |  |  |  |  |  |  | 1.06 | 1.03, 1.09 |  |  |  |
| Red meat | 100 g/day |  |  |  |  |  |  |  | 1.02 | 0.86, 1.21 |  |  |  |
| Energy from fat | 125 Kcal/d |  |  |  |  |  |  |  | 0.97 | 0.94, 1.01 |  |  |  |
| Energy other sources^c^ | 125 Kcal/d |  |  |  |  |  |  |  | 1.01 | 0.97, 1.05 |  |  |  |
| Physical Activity |  |  |  |  |  |  |  |  |  |  |  |  |  |
| (Moderately) Inactive |  |  |  |  |  |  |  |  | 1.00 | ref |  |  |  |
| (Moderately) Active |  |  |  |  |  |  |  |  | 1.00 | 0.93, 1.07 |  |  |  |
| Smoking status |  |  |  |  |  |  |  |  |  |  |  |  |  |
| Non-smokers |  |  |  |  |  |  |  |  | 1.00 | ref |  |  |  |
| Smokers^d^ |  |  |  |  |  |  |  |  | 1.19 | 1.11, 1.27 |  |  |  |
|  |  |  |  |  |  |  |  |  |  |  |  |  |  |
| *Aggregate level* |  |  |  |  |  |  |  |  |  |  |  |  |  |
| Dietary fiber | 10 g/day |  |  |  |  |  |  |  | 0.86 | 0.65, 1.14 |  |  |  |
| Alcohol | 15 g/day |  |  |  |  |  |  |  | 1.28 | 1.05, 1.55 |  |  |  |
| Energy other sources^c^ | 125 Kcal/d |  |  |  |  |  |  |  | 1.16 | 1.06, 1.26 |  |  |  |
| % Graduate | 1% increase |  |  |  |  |  |  |  | 0.99 | 0.94, 1.04 |  |  |  |
| % Smokers^d^ | 1% increase |  |  |  |  |  |  |  | 1.07 | 1.00, 1.13 |  |  |  |
| Latitude^f^ | 5° decrease |  |  |  |  |  |  |  | 0.94 | 0.90, 0.98 |  |  |  |
|  |  |  |  |  |  |  |  |  |  |  |  |  |  |

^a^ Variance component estimate; ^b^ Also adjusted for weight, height and educational status; ^c^ Energy from sources other than fat and alcohol; ^d^ current and former smokers; ^e^ log-RR scale; ^f^ Modelled as cosine(Latitude); ref=reference category.
